# Extracts and Gleanings from Our Exchanges

**Published:** 1873-11

**Authors:** 


### PART II.

### EXTRACTS AND GLEANINGS FROM OUR EXCHANGES.

### *MULTUM IN PARVO.*

A NEW METHOD OF USING ELECTRICITY; CENTRAL GALVANIZATION.—During the past two years and more, says Dr. G. M. Beard, (New York *Medical Journal*,) I have been gradually systematizing and perfecting a novel method of electrization, to which I have given the name *central galvanization.*

The results obtained by this method are so brilliant and important—in many cases over other methods are so decided—that I have thought best to here describe it in detail.

In central galvanization the negative pole is applied to the epigastrium (the patient holding it by an insulated electrode,) while the positive is applied over the head, around the sympathetic, and down the whole length of the spine, in such a way as to bring the brain, the pneumogastric, the spinal cord, and all the prominent plexuses of the sympathetic, indeed, the whole central nervous system, under the influence of the current. I began to work up the method of central galvanization as early as 1869, but did not fully systematize the method, in all its details and in its completeness, until the following year. The method, as I now employ it, is a growth from small beginnings—a result of long and various experimenting.

*Details of the Method.*—The negative pole is placed on the pit of the stomach, for the twofold reason that it is well borne there, and that a descending current seems to act better in most cases than the ascending. Whether the differential action of the ascending and descending currents is due to the *direction* of the current, or to the poles, I am unable to say. The positive pole is less sensitively felt than the negative, and is less irritating, and it is not unlikely that this fact may explain the more satisfactory results of the descending currents in central galvanization.

It will be seen that the reasons here given for generally placing the negative pole at the pit of the stomach are the same that we have elsewhere given for placing the negative at the feet or at the coccyx in general faradization. (Beard & Rockwell's "Medical and Surgical Electricity.)

In some systematic comparative observations that I made at Demilt Dispensary, the reverse method—placing the positive pole at the pit of the stomach—did not seem to be satisfactory, and similar experiments with the positive pole at the coccyx, or at the feet in general faradization, led to a like conclusion. The only way to determine a question of this kind is by comparing many applications on a variety of patients; in individual cases, no difference can be traced in the effects of the ascending or descending currents.

I do not always make the applications all over the head, but merely on the forehead, gently passing the electrode from one side to the other; then baptize the patient on the *cranial centre*, at the top of the head, and rest the pole there for about one minute, and sometimes longer. To the head I apply from two to six or eight cells — for patients vary in their susceptibility — and beginning with a weak current, and gradually increasing until a sour or *metallic taste* is perceived in the mouth. The *cranial centre* — the summit between the ears — I regard as the most important region of the head in all electrical applications, and especially in central galvanization. A current passing from that point to the epigastrium, traverses the centre of life — if life has any centre—and affects the sympathetic, and the roots of the facial nerves. The sensation produced by this application is different from that of any other application to the head, and is sometimes indefinable.

An application to this point for one or two minutes is usually about as much galvanization as the brain needs. In exceptional cases, where the hair is thin, or the head is bald, I make the applications all over the surface, back and front. In applications to the head, care should be taken to avoid sudden interruptions, or shocks that cause dizziness; the flashes of light before the eyes are of little account, but nothing is gained by producing them, and they are annoying to the patient.

The electrode is then passed down the inner border of the sterno-cleido-mastoid muscle, from the auriculo-maxillary fossa to the clavicle, for the purpose of affecting the pneumogastric and sympathetic. I usually make the application on both sides, and from one to five minutes.

In galvanizing the spine, especial attention is given to the *cilio-spinal* centre, below the first and seventh cervical vertebræ, which is to the spine what the cervical centre is to the brain. The cervical sympathetic and pneumogastric, as well as the spinal cord, are affected by the current. The electrode should also be passed the entire length of the cord by *labile* applications up and down. The back is not usually sensitive, and strong currents, from ten to thirty cells, can be borne without any more discomfort than a burning or pricking sensation beneath both electrodes.

The back may be treated from three to six minutes, and the whole length of the *seance* of central galvanization ranges from five to fifteen minutes.

*Preparation of the Patient.*—All the preparation a *male* patient requires for central galvanization is to unbutton the collar, remove the coat and vest, and slip off the whole clothing, so that free access can be had to the spine.

A *female* patient may remove her corsets and slip up her underclothing, or merely loosen the clothing at the neck and waist, so as to make room for an electrode to be passed down to the epigastrium, and for a spinal electrode to be passed up and down the back.

*Electrodes.*—For the negative electrode at the pit of the stomach any electrode with a broad surface, so as not to be too painful, and an insulated handle that the patient can hold, will answer.

For the positive pole, I prefer my *adjustable* electrodes, of different sizes. These can be passed under the clothing with great ease, and are wonderfully convenient for many of the purposes of electrization. They can also be provided with flannel covers, that may be washed as often as necessary.

*Central Galvanization compared with Localized Electrization of the Nerve-Centres.*—Before I had perfected the method of central galvanization, I had endeavored to fulfil the same indications for which it is required, by successfully localizing the galvanic current in the brain, the cervical sympathetic, the pneumogastric, and the spinal cord, by the usual method of galvanizing these parts. The results, though sometimes all that could be expected, are not very far inferior to those obtained from central galvanization, and for these two reasons: 1. In their localized applications, both poles are brought to bear on the different parts of the nerve centres, and the irritating effect of the negative is very frequently injurious. 2. The successive localizations in the nerve-centres are very inconvenient for the patient, and very laborious for the operator, since they require constant change of the position of the patient and of both electrodes.

*Central Galvanization compared with General Faradization.*—General faradization is the method of all others with which central galvanization would be most naturally compared, since it is used for very many of the same general indications as well as for the same special affections.

The leading difference between them is, that *central galvanization chiefly affects the central nervous system,* while in general faradization *a large part of the muscular surface of the body is acted on.* All other conditions being the same, central galvanization is differentially and specially indicated in those nervous diseases by whatever symptoms expressed, or by whatever name indicated, where, in spite of the nervous exhaustion or perturbation, the *muscular strength and general nutrition* are comparatively undisturbed. In many forms of aneuric disease, such for example as neurasthenia, hysteria, insanity, neuralgia, sick-headache, etc., the muscular development and capacity may not only be impaired, but *positively increased,* so that the patient can take very long walks and undergo a vast amount of physical toil without fatigue; such cases are most benefited by *central galvanization.*

On the other hand, when these or other diseases are accompanied or followed by loss of body weight, and by muscular flabbiness and feebleness, *general faradization* is indicated for the obvious reason that it is the most powerful method now known of developing the muscular system. In general faradization the central nervous system is, of course, affected, but to a less degree than in central galvanization. The practical difference between the two currents—faradic and galvanic—is mainly a difference of degree, due to the same difference that there is between bromide of potassium and hydrate of chloral, the faradic current being the bromide of potassium, and the galvanic the hydrate of chloral.

Bromide of potassium is a safer remedy than hydrate of chloral, fulfills a wider range of indications, but there are very many cases where it is powerless, and the hydrate of chloral acts as a specific; so the faradic current is safer than the galvanic, and therefore better adapted for general use, and, for those who use but one current, fulfills a larger requirement; and yet there are many cases when it fails and the more powerful galvanic is demanded.

It is this superiority of *degree* that makes the effects of central galvanization so much more positive and certain than faradization, however administered, in cases of nervous diseases of all kinds, and especially in gastralgia, angina pectoris, neurasthenia, and spinal irritation.

Central galvanization is indicated in very many of the same diseases as general faradization, but is differentially indicated in those conditions where the brain, spinal cord and sympathetic, pneumogastric and the large plexuses are involved. Thus in hysteria, hypochondria, insanity, gastralgia, angina pectoris, chorea, and spasmodic affections. nervous dyspepsia, where the system has not been greatly debilitated; in spinal and cerebral exhaustion, spinal irritation and congestion, and in certain diseases of the skin, central galvanization I have found, on the whole, more efficacious than general faradization. In some cases of hysteria, neurasthenia, anæmia, and in nervous dyspepsia when the weight of the body has been greatly reduced, and in general debility of various kinds, I have found general faradization, on the whole, superior to central galvanization. Some of the very best results have been obtained by *alternating general faradization with central galvanization.* In some cases it happens that a change from one method to the other is of great advantage. Some patients, with a strange caprice, act better under the one than under the other at one stage of their disease, whatever it may be, and at some other stage the conditions are reversed.

I have judged of these methods by the statements of the patients during the *seance* or directly after the *seance,* in the intervals and at the end of a course of treatment. I have judged by the appearance of the patients, by their changes in weight, size, and color, by the variations in the pulse, the temperature, the general circulation, the vascularity of the retina, by the relief of pain, the improvement in sleep and digestion, by the increased capacity for muscular and cerebral toil, and by the local effect of the tissue as manifested to the ear of the observer. (Dr. Merideth Clymer informs me that, for the year past he has been in the habit of directing the galvanic current through the central nervous system by a method substantially the same as central galvanization. It is a fact of interest that, although he arrived at this method later than I, he yet devised it entirely independently, and used it for many of the same indications and with equally satisfactory and remarkable results.)

In general, it may be said that *powerful stimulating tonic effects* are produced by both methods. The improvement in sleep and appetite, and in capacity for muscular and mental toil, and exhilaration, temporary and permanent, are observed either at the close or in connection with central galvanization, as after general faradization. *Increase of body-weight* is, I think, more marked after general faradization.

On Post-partum Dietetic Treatment.—Dr. Thomas Cairns, in an article published in the "Transactions of the Edinburgh Obstetrical Society," lays down three rules of diet, vis:

1. "The diet should be nutritious in point of quality.
2. "It should be small in quantity and frequently repeated.
3. "It should be varied in kind and form."

He says of the usual low-diet system, "Such treatment, I humbly maintain, is utterly inconsistent with the principles of obstetrical pathology. I wish I had time to inform the younger members of the Society the real meaning of the term *labor*, because I am certain, if they knew what it actually expresses, they would at once perceive the necessity, on purely pathological grounds, of *feeding* and not *starving* their puerperal patients. Consider the waste of tissue to which the woman is subjected during her confinement, and you must see that unless that waste be supplied by proper and fresh materials, the patient's strength must necessarily be very much reduced below its normal condition; and should any untoward event happen to her in these circumstances, the prognosis must essentially be infinitely more unfavorable than if her system had been duly sustained by generous diet. On these grounds I have always been in the custom of liberally administering to my puerperal patients the most nourishing food which their circumstances enable them to procure, such as soft-boiled eggs, beef-tea, soups, chops, steaks, tripe, etc., with a glass of wine daily in addition, or if the patients prefer it, a glass of ale or porter; and this treatment, I humbly aver, is based on sound pathological principles.

Let the food be administered in small quantities at a time, and at such intervals as shall have insured the complete digestion of the previous diet. Common sense seems to suggest that with the view of stimulating the appetite and imparting to the patient a positive relish for food, every advantage should be taken of the culinary art in dressing the same article in different forms, and when these have been exhausted, that one article should be substituted for another during the whole period of convalescence.—*Phil. Med. Times.*

Clinical Lecture on Eczema Rubrum.—Dr. Louis Duhring, in delivering a lecture on skin diseases at the Dispensary, April 4th, 1872, said:

*Gentlemen:* The patient before you presents one of the commonest, and, because one of the commonest, therefore one of the most important and interesting diseases of the skin which you are likely to meet.

He is a boiler-maker by occupation, of temperate habits, and 41 years of age. His trouble began about six months ago, after he had received a scratch from a nail, on the front of his leg. It soon itched, and later became wet and discharged a fluid. At present the limb is painful when he is obliged to stand upon it. Such is the history which we gain from the patient. Now let us see what the appearances are.

We have upon this leg a disease of six months' standing, distinguished by a good deal of chronic inflammation of a certain character. In places the skin is denuded of epidermis—raw, in fact—and you can see the corium with the torn apices of the papillæ. On close examination numerous small vesicles, the size of a mustard-seed, can be seen here and there, many of them broken open and torn.

There is, and has been, much itching, and the patient cannot resist the temptation to scratch. I might observe here, however, that in a certain number of cases of eczema rubrum, itching is not so prominent a symptom as in this case, though both itching and burning are a general rule.

The general appearance of the diseased skin presents a dusky red color, with the bases of the numerous torn vesicles indicated by bright red points. From the greater portion of the surface there is a continual oozing of a pale, yellowish, sticky, syrupy fluid, which accompanies no other disease of the skin.

After a few hours' application, a bandage or stocking covering this limb will become wet through and through, and then drying, will become stained a light straw-color, and stiffened as if with sizing.

Over some portions of the limb you will observe that the appearances are different; for here we have no redness nor crusts.

Let us examine closely these various manifestations, and see what we can learn of the history and nature of the affection we are studying.

The direct causes of eczema are various. Excessive heat, blows, exposure to the weather, neglect, and dirt are some of these, and the varied appearances which the disease often presents are in some degree connected with the immediate cause.

In the case before us, we see scattered over the surface the vesicles of which I have just spoken. These are the earliest manifestations of the disease, and as we have seen, they are still making their appearance in some places. These vesicles are the seat of great and characteristic itching, the result of which is that the vesicles are torn open by scratching.

But this is not the only result of scratching, for the epidermis is abraded, and the corium also exposed, from the bruised papillæ of which blood issues forth to color the crusts of dried serum.

Another manifestation which we must not lose sight of in the study of this case is the crusts which cover some portions of the limb.

If you will consider a moment, you will perceive that these crusts are simply the dried serous exudations, which, were the leg altogether neglected, would cover it entirely, but which can always be prevented from forming with proper attention and treatment.

Let me call your attention to a cause of the disease we are considering, in the shape of these varicose veins you perceive over the surface of the leg.

Varicose veins, especially in women, are a frequent cause of eczema rubrum. They are often accompanied by itching, which is most severe when the person is warm in bed, and during sleep the patient will scratch and tear the skin in the effort to get relief. In individuals who have a constitutional tendency to eczematous eruptions; this scratching is sufficient to bring on an attack of the disease; and there is little doubt but that these enlarged veins in this man's leg have contributed materially towards bringing on the condition before us.

The disease, as you observe, is confined to one leg. Eczema rubrum is an affection which may or may not be symmetrical in its occurrence; it may attack one limb or both.

We have here, then, a case of eczema rubrum, or as I perfer to call it, eczema madidans, from the characteristic weeping or oozing of serous exudation.

This affection is not usually self-curable, and if allowed to go on without proper treatment may last indefinitely, the area of disease extending, and the infiltration of the tissues becoming more and more marked. We often see eczema rubrum of the leg, of twenty years or even longer duration. Nevertheless, when taken properly in hand, with an appropriate course of treatment instituted, and the directions of the physician faithfully carried out, the disease is one of the most curable of all skin affections, and when once cured is not likely to relapse.

Now, as regards the treatment. You must remember that this case is one of chronic disease, and that the treatment which is highly appropriate here, would not suit at all in an acute eczema of a few weeks' duration. I shall order for this man two to four ounces of sapo viridis and eight ounces of unguentum diachyli.

The former is simply a potash soap, which is manufactured in Germany. The unguentum diachyli is not so well known, and you will find the formula for its preparation in the University of Pennsylvania Pharmacopœia, and in Neumann's Hand-book of Skin Diseases.

The method of using these remedies is as follows:

The affected limb is first rubbed smartly for some minutes with a flannel rag covered with the soap, the rubbing being so brisk as even to make the skin bleed a little. Then, the soap having been washed from the surface by warm water, and the limb dried by gentle patting—not rubbing—with a soft towel, the ointment is applied, spread quite thickly on strips of soft linen about three inches wide and sufficiently long to go entirely around the leg, thus covering the diseased portion completely.

The effect of the rubbing with soap is at first to cause quite a good deal of stinging and burning, and the leg for the time looks worse than it did before.

The application of the ointment, however, soon soothes the part materially, and great comfort is experienced for the next ten or twelve hours. At the end of that time the itching has set in again, and it is time for a new application of the soap and ointment. You should, if possible, see your patient the very next day after ordering this treatment, in order to ascertain whether your directions have been complied with.

You will almost invariably find that some mistake has been made, for it is rare to find a patient who will at first carry out every detail of the plan, and unless everything is done properly, you will not get a satisfactory result.

This treatment should be continued, the rubbing with the soap and application of ointment being made with the same amount of care. At the end of six weeks a very decided change for the better will be observed. The vesicles will cease to form, the serous oozing will no longer be noticed, the congestion and infiltration will have disappeared, and the skin will have once more assumed a healthy appearance.

McIntosh on Dysmenorrhœa and its Treatment with Sulphate of Quinia and Extract of Stramonium Seeds.—The results of an experience with each of these drugs, used separately, led Dr. McIntosh (American Quarterly Journal of Medical Science, Jan., 1873,) to unite them in the following proportions, varied according to the requirements of each individual case: "Give a pill consisting of one-fourth to one-third grain ext. daturæ stramon. sem.; one-half to three grains sulph. quinia; one-fourth to one-half grain opii; one to two grains camphor, three times a day for five days, beginning three days before the catamenial discharge, and continuing for two days after its inception. The same treatment is to be commenced just previously to the next monthly period; and usually from four to eight repetitions, where there is no mechanical obstruction, will secure a regular, painless monthly flow." Latterly Dr. McIntosh has added powdered ipecacuanha to the above pill, and, as he states, "with benefit." With the foregoing treatment should always be combined such emmenagogue and furruginous medicines as an anemic or other condition may require, while special directions should be given to procure a daily action of the bowels. A careful avoidance must be observed of exposure to cold or wet, and great care in keeping the feet warm, and a good circulation in the lower extremities generally.—*Medical Record.*

Feeding by the Nose.—The ease and efficiency of this method of introducing food is commended by several correspondents of the *Lancet.* Medicine may be administered in the same way. Joseph Westmoreland writes: "I have often thought food might be administered through the nostrils with less trouble to the surgeon and greater ease to the patient than by the method with the stomach-pump through the mouth. There are some cases of scarlet fever in which this method may be very useful. At different times in the wards of the Fever Hospital connected with the Manchester Working Hospital I have had recourse to this plan of feeding patients suffering from scarlet fever when the tonsils and parotid glands have been so much inflamed and enlarged as to render deglutition most difficult, if not impossible. In such cases I have taken a gum-elastic catheter, to one end of which I fitted a piece of caoutchouc tubing about two feet in length, such as is ordinarily used for children's feeding-bottles, the other end of the tubing being fitted to a funnel. I then instructed the nurse how to pass the catheter along the floor of the nose into the pharynx, the nurse then having nothing to do but gently pour the beef-tea, milk, or other aliment into the funnel. I have had scarlet-fever cases fed in this manner with both ease and benefit to themselves; at the same time, however, I had the mouth and throat kept moist by administering frequently a teaspoonful or two of cold water by the mouth. I have also had a typhus case fed in this manner, who violently resisted the administration of food in the mouth, and persistently spat it out when given."

W. E. Humble, M. D., London, states: "In January, 1871, a strong middle-aged woman, who had been for some years in a lunatic asylum, but had been discharged, again became insane. Among her delusions and insane acts she determined on starving herself. I endeavored by arguments to induce her to take food, but to no effect. I assured her I would give it by force, but she still refused. Having waited as long as I thought right, I had a pint of good beef-tea made. She was laid on her back on a bed, her head held securely, and I, resting my hand firmly on her cheek to guard against any sudden movement of the head, inserted the nozzle of a small funnel into one nostril, and poured with the greatest facility by tablespoonfuls the pint of beef-tea into her pharynx. Of course involuntary deglutition occurred with each spoonful, and the perfect ease with which the food was given, and the absolute impossibility of preventing deglutition, not only surprised, but much amused the bystanders. It would, doubtless, have answered the same purpose to have introduced the nozzle of a gutta-percha funnel or other like instrument into the mouth sufficiently far to pass over the back of the tongue, or to adopt any other means of passing the food beyond the physiological limit of voluntary deglutition, but it would have been less convenient. The plan I adopted was suggested by an article in the "Half-yearly Abstract," by Dr. Moxey. What occasion can there be under ordinary circumstances for a stomach-pump to introduce food into the stomach? Once the food introduced into the pharynx—pharynx and spinal chord being healthy—the œsophagus is a far better tube than the tube of a stomach-pump, and no injury is done."

Alex. W. Macfarlane says: "I have read with interest the correspondence on the above subject, of which my experience is limited. There is one point, however, which I deem of much importance, and which seems to have escaped notice, and that is, the volume in which the fluid is given. When given slowly and in small volume, the patients seem to be able to control it to some extent, and spit it out by the mouth; whereas if given in larger volume and hurriedly, the sense of impending suffocation makes them gulp it down. As to the mode of administration, I have tried a large syringe, small funnel, bottle, and an ordinary feeding cup, and, attending to the above points, all answered equally well."—*Medical Cosmos.*

Treatment of Small-Pox by Baths.—At a recent meeting of the Dublin College of Physicians, Dr. H. Benson called attention to a form of treatment so prominently brought before the Society on a late occasion by Dr. Stokes. He referred to the treatment by the bath. He was so struck by the result in Dr. Stokes' case that he determined to adopt the treatment in the next suitable case he met. In a very few days such a case presented itself. The patient was a gentleman residing in one of the surburbs of Dublin. He suffered from an extremely bad form of confluent small-pox. It was remarkably confluent, not only on the face, but also on every part of the body. The pustules were not well filled, but were flat, and the face presented the appearance as if a wax candle had been dropped over every part of it. During the secondary form the delirium became exceedingly troublesome, and the patient quite uncontrollable. For the previous twenty-four hours he had not been in bed for five minutes, and he had had no sleep for over thirty-six hours. Hypnotic remedies had no effect, and it was not possible to apply leeches or other applications to the head. With some difficulty he was placed in a slipper-bath, of the temperature of 98 deg., and he immediately exclaimed "its glorious, its delicious, its delightful." He became at once calm, collected and obedient, and within fifteen minutes he ceased to have any delirium. After half an hour, he slept in the bath for two hours, occasionally waking for a minute or two while fresh water was being added. He—Dr. Hawtrey Benson—kept the patient in the bath for five hours and a half, removing him after that on account of headache which supervened. He was then put to bed, perfectly free from delirium, and with the help of fifteen grains of chloral (of which four times that dose had no effect previously) he slept uninterruptedly for eight hours. The case progressed from that out without the slightest check. Dr. Hawtrey Benson thought that this treatment did not receive its due measure of attention at the hands of the profession. Dr. Grimshaw asked what was the temperature of the man's body when he went into the bath.

Dr. H. Benson replied that the temperature could not have been lower than 104 deg., and the bath was at least six degrees lower.—*Canada Medical Record.*

DIPHTHERITIC ALBUMINURIA.—R. Browning, L. R. C. P. L., in the British Medical Journal says:

From what I have lately witnessed while watching two local epidemics of diphtheria, I am disposed to consider that albuminuria is present in nearly all cases. That its appearance is usually about the end of the first week after the diphtheritic membrane is developed, though sometimes earlier more rarely later. Coexistently with its appearance, there is a notable diminution of the quantity of urine, and an increased excretion of urea; whilst lithates generally, tube casts, both granular and waxy frequently, blood corpuscles not seldom, and pus globules occasionally are found on examination of what is secreted. The urinary specific gravity mostly averages 1016, and the temperature of the body is, as a rule, 100.4 to 102 degrees.

The gravity of the prognosis increases in an equal ratio with the quantity of albumen existing in the urine, independent of the amount of throat affection or kidney disorganization, and an early or late discovery of albumen is of serious import. The local mischief attacking the pharnyx, larnyx or other structures, and paralysis sometimes occurring are entirely the result and symptomatic of a morbid poison affecting the general system, just as the sore throat of syphilis is the sequence of a blood disease previously contracted. Albuminuria in any quantity, is due to obstruction of circulation through the kidneys, caused by congestion of the malpighian tufts, this congestion being produced by paralysis of the nerves supplied to them; but a mere trace only of albumen arises either from pus or else blood which has casually entered the volume of urine. The indication of treatment is to remove this obstruction by overcoming the paralysis, and this is best accomplished by local faradization. Seven cases are reported in detail, two of which terminated fatally. In these two, no farizadation was employed. The other five, which were all of a very serious nature, recovered after faradization had been resorted to. All were marked by unmistakable evidence of blood poisoning and albuminuria, with more or less suppression of urine. The treatment of all was conducted on the same principles, plus or minus the induction coil; the object aimed at being at first, during the premonitory symptoms, to regulate the secretions, and then to support the strength of the system in every possible way. My sheet anchor was the tincture of perchloride of iron, sometimes combined with glycerine, sometimes with chlorate of potash, and sometimes given *per se.* Stimulants and nourishment in every variety were supplied with no sparing hand. The customary topical medication was of course attended to. In some instances, the ordinary conductors fitted to most galvanized batteries; in others, "Etna's" were employed. Faradism was thus employed over the lumbar regions along the lower part of the spine, and as nearly as possible in the direction of the ureters.—*Detroit Review of Medicine.*

In Prussia it requires 51 years for the Christian population to double itself, and only 41 years for the Jewish.

Diphtheroid Tonsillitis.—Dr. E. Harvey, of Chester, Delaware Co., (*Trans. of Pa. State Med. Soc.*,) gives this name to a disease characterized by the following symptoms: "The patient is chilly for a day or two, with slight soreness of the muscles. This is followed by a sharp headache, which is felt chiefly through the temples, then a stiffness and soreness of the muscles on the back of the neck, accompanied by pain in the legs, and sometimes numbness in the arms. While these general symptoms are coming on, the throat becomes sore, and this is what the physician's attention is called to. At first the tonsils, uvula, and a small part of the palate are red and swollen. In a day or two, yellow patches are seen on the inflamed tonsils, and are sometimes mistaken for ulcers. The general symptoms will continue a week or more if not treated, the chilliness gives way to fever, the pains increase, the throat becomes painful, deglutition is difficult, the patient is greatly debilitated and the debility continues for a week or two after the symptoms have disappeared. If taken in the early stage, this disease can be cured in about a day by chlorate of potassa, given in ten grain doses every hour for five or six hours, and afterwards every two or three hours while needed. It is often mistaken for diphtheria, sometimes for membranous croup by the inexperienced, and sometimes for quinsy when it is not seen and successfully treated until the throat becomes very bad. The disease is contagious, and generally affects all, or nearly all, of a family where it occurs. It attacks more than once the same person, and no season is exempt from it."

Berberis Vulgaris in Malarial Diseases.—Ague, malaria, and all kindred affections, cause enlargement of the spleen, and, if frequently recurrent or protracted in their course, enlargement of the liver also. Piorry, professor of pathology at Paris forty years, showed by a series of measurements that frebrifugal remedies act as such upon fevers by reducing the spleen and all other (sympathetically) enlarged viscera to the normal dimensions. Quinia had been recognized as possessing this reducing agency by Bally, Piorry's master, fifty years ago; but it was reserved for the pupil to show that the same virtue resides in the "berberis vulgaris," or common barberry, an *echantillon* of which plant, pharmaceutically prepared, had been given him by a chemist of Lyons. Piorry came to prefer this preparation in the treatment of miasmatic fever to quinia; indeed, whenever he found the spleen enlarged in a patient suffering from ague, intermittent, or hectic, he gave (as one of his pupils and clinical clerks, Dr. L. M. Klien, tells us) berberis instead of quinia, and the fever abated in a few hours. From comparative experiments of both febrifuges, Piorry convinced himself that in well-recognized cases of ague and miasmatic fevers berberis vulgaris was the superior remedy. He sent Dr. Klein to Algeria to institute further experiments, and the result was strongly confirmatory of Piorry's practice, which the physicians of the colony have subsequently adopted. The plant is a very accessible one, very common in almost every rocky upland in Europe, Asia, and America.—*Lancet.*

KALI BICHROMICUM IN DIPHTHERITIC CROUP—Dr. Ludlam remarks:

1. "This remedy seems especially appropriate to pseudo-membranous lesions of a diphtheritic nature affecting the respiratory mucous surfaces, as the nares, the superior portion of the pharynx, the larynx, the trachea, and the bronchial tubes, even down to their ultimate ramifications.

2. "Where the deposit is of firmer texture, more apt to be developed into casts which are cartilaginous, or pearly in appearance, elastic, fibrinous, and more securely attached to the subjacent integument.

3. "It is indicated in all those cases where a transfer of the local disorder to the larynx or trachea impends, as shown by soreness of the larynx when pressed upon from before backwards, aphonia, croupy inspiration or cough, and a desire on the part of the patient to lie with the head thrown far backwards in order to open the glottis.

4. "It may also be given with excellent results in case the tonsils are almost or quite enveloped by a thick and well-organized deposit, and in which at the same time the patient has an almost incessant cough.

5. "Also where, with the foregoing symptoms, there is an evident tendency to ulceration and deposit upon remote mucous membranes, as, for example, those of the uterine system. In my own experience, the Bichromate is almost a specific to diphtheritic formations upon the free uterine, and to those found upon the respiratory epithelial surfaces.

6. "Since in all these cases the putrid symptoms are less marked than in the pharyngeal and alimentary diphtheria, you should take the hint to cease the employment of Bichromate when these symptoms ensue. The Iodide of arsenic, Nitric Acid, or Carbo Vegetabilis, are much more decidedly indicated for the relief of such a condition."

Dr. Lord advises the administration of this remedy by inhalation. The following is an extract from his report of a very severe case in which the remedy caused an aggravation even when given in moderate doses.

"When the Bichromate was given at intervals of an hour or more, the patient uniformly got worse. The cough was almost constant, except in the night, when asleep. It ran up from a slight hacking to suffocation, which was only prevented by a means which I have purposely omitted to mention that I might direct your attention more particularly to it. After the 20th day, when the cough became dry, and the respiration whistling, and when suffocation seemed eminent, inhalations of Bichromate were used with prompt relief; of course it was only temporary, but it was a respite. But for it death must have ensued. It did not fail in a single instance of easing the breathing and loosening the cough, and ejection of membrane or large quantities of stringy mucus followed. The method was simple. Two or three grains of Bichr. 2 were put into a small tin teapot, and a half a teacup of hot water poured on. The vapor passing from the spout was inhaled. I do not think that any medicines given in this case but the Arsenic and Bichromate had any good effect. I was so well satisfied of this, that in all subsequent cases I have trusted entirely to the Bichromate as the specific remedy, and have had no reason to repent it. Other remedies may be required, but that is the remedy.

SUBCUTANEOUS INJECTION OF ERGOTIN IN VARIX.—Having observed the records (the British Medical Journal, April 27, 1872, from the Berliner Klin. Wochensch., March 4, 1872,) of good results from the subcutaneous injection of ergotin in cases of aneurism in the hands of Von Langenbeck, Schneider, and Dutoit, Dr. Paul Vogt, of Greifswald, was led to try the remedy in varix of the lower limb.

The first patient on whom the experiment was made was a man aged sixty, who had suffered for several years from extensive varices of the leg. A solution of two grammes of ergotin was made in 7.5 grammes each of spirit of wine and glycerine, and a quantity containing 12 centigrammes of ergotin was injected at the proximal end of a varix more than two inches long, and as thick as a little finger, lying over the tibia. The injection was repeated every second day. In eight days the varix could not be seen, and in six weeks no trace was left. During the treatment, the patient went about as usual. Another varix, of the size of a hazel-nut, lying on the outer side of the calf, was treated in a similar manner, with the same result. At the point where the injection was made there was some circumscribed infiltration, which gradually disappeared.

Several other patients in the Greifswald Hospital, some of them with very large varices, have been treated with the subcutaneous injection of ergotin, with a surprisingly good result.

Dr. Vogt believes that the ergotin causes contraction of the muscular coat of the arteries, so that the flow of blood into the dilated vessels is hindered; that it also produces contraction in the veins; and that the local infiltration following the injection may have some effect by the compression which it exercises.—*Phil. Med. Times.*—*Nash. Med. Journal.*

BLISTERS IN PNEUMONIA.—Dr. C. J. B. Williams, the distinguished English authority, makes use of the following language in speaking of the treatment of pneumonia: "My experience has taught me to put great faith in large blisters, both in asthenic pneumonia and in bronchitis, and I am confident that I have seen many lives saved by their means. Instead of being lowering, they give a salutary excitement to the circulation, and the copious serous discharge which they produce from the skin, tends to relieve the congested lung without wasting the red blood that is so needed to sustain the functions. Small blisters tease as much as large ones, and are far inferior in the relief they afford."—*Medical Press.*

CAFEINE (*Lancet*, Oct. 12).—In a recent number of *Pfluger's Archive*, Dr. Aubert describes some researches on this substance. Seeking to ascertain the proportion of cafeine or theine in a cup of coffee or tea, he arrived at numerical results somewhat above his predecessors. According to him, a cup of coffee, forming an infusion of 16.75 gr. of dry coffee-grains, contains about 0.1 gr. to 0.12 gr. of cafeine; and an infusion of 5 gr. to 6 gr. of dry leaves of very good tea contains about 0.1 gr. to 0.12 gr. of cafeine.

He studied the effect of this alkaloid on the nerves, muscles, respiratory movements, heart, and circulation. Cafeine increases the reflex excitability, and may produce tetanus. Dr. Aubert, with most authors, considers this a medullary tetanus; for it is not produced in the leg of a frog if the ischiatic nerves are cut, and it takes place in a limb the circulation in which has been stopped by a ligature before the subcutaneous injection of the cafeine into the skin of the back. An injection of 0.005 gr. into the skin of a frog, 1.20 gr. into the jugular of a rabbit, 0.200 gr. into the jugular of a dog or cat, produces tetanus. Dr. Aubert did not observe the weakening of excitability of the nerves referred to by Voit and others. According to him, the nervous excitability is altered only in the case of the nerve being plunged directly into a solution of cafeine. The muscular excitability is not affected so long as the cafeine is not applied to the muscles themselves.

Confirming Uspenky's experiments, Dr. Aubert shows that the production of apnœa by means of artificial respiration (?) counteracts the development of the convulsions produced by cafeine,—a phenomenon similar to that which Rosenthal was the first to apply in cases of strychnic tetanus, and which appears to be applicable to all tetanus produced by reflex influence. As to the dose necessary to kill an animal apnœised, they were various: 3 gr. of cafeine did not kill a dog of 10 kilos. in which artificial respiration was produced. Other dogs in the same condition succumbed to an injection of 0.25 gr. of cafeine. Cafeine at first produces an increase in frequency of the pulse, but a diminution of its bulk takes place very quickly (one minute after the injection), and sometimes determines the imediate death of the heart. Small doses, 0.1 gr. to 0.15 gr., injected into the skin, produce no effect on the heart of a rabbit, while a dose of 0.25 gr. causes an acceleration of the heart and respiratory movements. The increased rapidity in the heart-beats and the rise of arterial pressure observed may be attributed, Dr., Aubert thinks, to a paralysis, more or less complete, of the nerves proceeding from the ganglia to the muscles of the heart and an excitation of the arresting apparatus of the heart. He does not agree with those who say it is cafeine which gives to coffee its principal qualities. He thinks the reviving action of coffee, which makes it such a favorite beverage, is not yet scientifically explained.

POPULATION OF GERMANY.—Including Alsace and Lorraine, the subjects of the Kaiser number, according to the census of December, 1871, 41,958,000. The females are in excess of the males by 768,000.

Carbazotate of Ammonia to Supersede Sulphate of Quinine (*Ib.*).—In a recent communication to the Societe de Therapeutique de Paris, Dr. Dujardin-Beaumetz investigated the character, properties, and uses of carbazotate of ammonia (combination of ammonia with carbazotic, picric, or trinitrophenic acid), especially as a therapeutic successor to sulphate of quinine. After relating the successful employment of this salt in intermittent fever by Braconnot, Calvert, Aspland, Bell, Chazereau des Thureaux, Manopa, etc., Dr. Beaumetz gave the results of six cases treated by himself, and of various experiments carried on upon animals and man. Like quinine, carbazotate of ammonia diminishes the strength of the pulse, and brings on heaviness, cephalalgia, and even delirium, and is eliminated by the kidneys. These effects have again been stated by Dr. Beaumetz. As to the clinical results they may be summed up thus:—Case 1: Quotidian ague; recovery after four days' treatment; daily dose from one to two centigrammes of the substance in pills. Case 2: Quotidian ague (sulphate of quinine having been given without effect); complete recovery after five days; five pills used. Case 3: Tertian ague; recovery after eight days; two pills a day. Case 4: Quotidian ague; recovery after eight days. Case 5: Facial neuralgia; speedy recovery. Case 6: Tertian ague; sulphate of quinine had been administered seventeen days with no result; completely cured after the administration of six centigrammes (about one grain) of the salt for two days.

Dr. Beaumetz draws the following conclusions from the various facts observed in his six cases or brought out by his experiments: Carbazotate of ammonia is very efficacious in intermittent fever; the suppression of the paroxysms may be obtained by the use of two to four centigrammes (one-third or two-thirds of a grain) daily; given in these doses the drug has never had any bad effects, and seems to be better tolerated than sulphate of quinine; the physiological action of the substance closely resembles that of sulphate of quinine.

Warm Salt Baths in Infantile Fevers.—The difficulties which attend the use of cold baths, when it is desirable to reduce the temperature in very young children, has led Schwalbe, of Zurich, to try warm baths. His patient was a delicate rachitic child suffering from catarrhal pneumonia, and but little over a year old.

The baths contained from 3 to 5 per cent. of common salt, and were given at a temperature varying between 86 deg. and 88 deg. Farenheit. At first they were employed from once to three times daily. The child made a good recovery. Many months after a similar attack occurred, and the same treatment was adopted, with the exception only, that the baths were more frequently repeated. The child made a favorable recovery the second time. It was observed that after each bath the temperature of the body was reduced from 2 to 4 degrees.—*Memorabilien*, 1872.

The largest number of deaths from scarlet fever in England from 1866 to 1870 was in 1870, when 32,543 died.

Borax and Nitrate of Potassa for Loss of Voice.—Dr. John W. Corson, of Orange, N. J., in the N. Y. Medical Record of January 1, 1873, gives a number of cases, and then says:

For clearness and convenience, we may sum up the results of our experience in the following brief conclusions:

1. That in sudden hoarseness or loss of voice in public speakers or singers, from "colds," relief for an hour or so, as by magic, may often be obtained by slowly dissolving and partially swallowing a lump of borax the size of a garden-pea, or about three or four grains, held in the mouth for ten minutes before speaking or singing. This produces a profuse secretion of saliva, or "watering" of the mouth and throat. It probably restores the voice or *tone* to the dried vocal cords, just as "wetting" brings back the missing notes to a flute when it is too dry.

2. Such "colds" may be frequently "broken up" at the very commencement; and this restorative action of the borax to the voice may be materially aided by promptly taking, the evening previous to a public concert, dissolved in a glass of sweetened water, a piece of the nitrate of potassa, or saltpetre, a little larger than a garden-pea, or about five grains, on going to bed, and covering with an extra blanket. The patient should keep warm next day. This both moistens the dry throat and further relieves the symptoms of "cold" and slight blood-poisoning from suppressed perspiration, by reopening the millions of pores of the skin more or less closed by cold.

3. These remedies have the three recommendations of being easy to obtain, convenient to carry in traveling, and perfectly harmless.

4. They are nearly or quite useless in the actual cure of long-continued chronic disease of the throat, or acute inflammation or "tonsillitis," both of which require other appropriate treatment.

The Oxalate of Protoxide of Iron.—At a recent meeting of the Academie de Medecine, (Bull. de l'Academie, Oct. 12,) M. Caventou delivered in his report on this substance, which had been laid before the Academy two years since by M. Girard. The reporter stated that the mode of preparation recommended by M. Girard is in no wise different from that usually employed in laboratories. Its therapeutical properties have been carefully tested by M. Herard in cases of chlorosis and anæmia, and these prove to be worthy of attention, presenting a preparation of iron which, while proving efficacious, has no tendency to produce constipation. The preparation is almost insipid, is readily taken by patients, and easily borne by the stomach. Given in doses of from ten to twenty centigrammes per diem it increases the strength and cures chloroanæmia as well as other good preparations of iron, while it establishes a peculiar claim by not causing constipation. Indeed, by raising the quantity to from thirty to fifty centigrammes, an aperient action is obtained. M. Caventou considers that this remedy should be indorsed with the recommendation of the Academy, which is necessary for the authorization of new remedies during the intervals that elapse between the editions of the Codex.—*Med. Times and Gaz.*, Nov. 23, 1872.

Catarrh.—Dr. Noyles said he was much pleased with the action of stimulants in acute catarrh. He never knew them to fail, though, for obvious reasons, it was not wise to recommend them to all. Other sedatives, as quinine or opium, accomplished the same end much more safely. The treatment of chronic catarrh by nitrate of silver has been extensively used and abused. Dr. Green, of New York, is said to have passed probangs wet with strong solutions (40 to 80 grains to ounce) into the trachea. He had passed the probang himself, but it was a difficult operation. As in the eye, nitrate of silver excites a new inflamation in the follicles of the throat, which carries off the neoplastic formation. In his specialty he often was required to treat the results of chronic catarrh, for it may extend to the Eustachian tube—the middle ear—to the formation of abscess, or even perforation of tympanum. He had tried all methods, douches, powders, vapors, etc. The douch needs to be used with caution, or the Eustachian will be flooded, and inflamation of the middle ear ensue. This method is especially unsafe for the laity to use.

Dr. Gilbert said that it was very important to perfectly cleanse the membrane ere making any application to it. A favorite local remedy with him was chloride of zinc, one grain to the ounce. Constitutionally he gave stillingia, combined with iodide of potassium.

Dr. Carstens has tried all the local applications for chronic catarrh, but found none so efficient as glycerine. Internally he used a combination of iodide of potassium and chloride of ammonium.

Dr. Jenks said that in acute catarrh he prescribed small doses of morphia (gr. 1-20 to 1-30) with great satisfaction.—*Detroit Academy of Medicine.—Detroit Review of Medicine.*

Hypodermic Injections of Quinia.—The salts of quinia, and especially the hydro-chloral, says Dr. Otto, are not sufficiently soluble to be commonly employed in subcutaneous injections, and the surgeon can neither be sure of the dose required, nor of the rapidity of the action. He recommends the use of pure quinia dissolved in ether; this solution is much less irritating than either the acid or alcoholic solutions. Quinia dissolved in ether in sufficient quantity to produce a prompt action, and to permit a considerable dose to be injected. The quinia should be dissolved in the ether, which should then be filtered and allowed to evaporate to some extent, so that a more concentated solution may be obtained. The solution he uses contains, in about half a drachm, five grains of sulphate of quinia. Dr. Otto has never observed any local inflammation caused by the injection of this solution, and he has injected as much as five grains of the quinia at one time without finding any other inconveniences than those which ordinarily accompany large doses of quinia, such as buzzing in the ears. The injection of this quantity rapidly produces a depression of the temperature of the body amounting to 1 deg. C. Hypodermic injections of quinia are particularly suitable to cases of puerperal fever, and those of purulent infection; but they may also be employed with advantage in cases of intermittent fever.—*Practitioner*, Sept., 1872.

